# Vitamin B5 copper conjugated triazine dendrimer improved the visible-light photocatalytic activity of TiO_2_ nanoparticles for aerobic homocoupling reactions

**DOI:** 10.1038/s41598-024-52339-2

**Published:** 2024-02-01

**Authors:** Samira Zamenraz, Maasoumeh Jafarpour, Ameneh Eskandari, Abdolreza Rezaeifard

**Affiliations:** Catalysis Research Laboratory, Department of Chemistry, Faculty of Science, University of Birjand, Birjand, 97179-414 Iran

**Keywords:** Materials chemistry, Organic chemistry, Supramolecular chemistry, Catalysis, Heterogeneous catalysis, Photocatalysis

## Abstract

In this work, Cu-vitamin B5 (pantothenic acid) bonded to 2,4,6-trichloro-1,3,5-triazine produced a bioconjugated dendrimer giving rise to the visible-light photocatalytic activity of nanocrystalline TiO_2_. XPS spectra uncovered the coexistence of Cu(II)/Cu(I) oxidation states with a predominant contribution of Cu(I). The new heterogeneous bio-relevant Cu-photocatalyst (Cu(I) Cu(II) [PTAPA G2-B5] @TiO_2_) revealed a band gap value [E_g_ = (2.8 eV)] less than those of Cu free components [PTAPA G1-B5]@TiO_2_ (3.04) and [PTAPA G2-B5]@TiO_2_ (3.06) and particularly the bare TiO_2_ (3.15 eV). The reactions showed to be light-dependent with the best performance under room light bulbs. The photocatalytic efficiency of the as-prepared heterojunction photocatalyst was exploited in the aerobic C_sp_^2^–C_sp_^2^ homocoupling of phenylboronic acid and C_sp_–C_sp_ homocoupling of phenyl acetylenes under visible-light irradiation to prepare structurally and electronically different biaryls. A radical pathway relying on the photogenerated e− and h+ and involving the Cu(I)–Cu(II) synergistic cooperation was postulated. The reusability and stability of the catalyst were verified by the recycling test, FT-IR spectra, and ICP-OES analysis.

## Introduction

The semiconductor-mediated solar- and visible-light-driven heterogeneous photocatalysis has been known as a low-cost high impact technology for energy, conversion, environmental remediation, and organic synthesis^1–4^. Among the available semiconductors, TiO_2_ as an inert and safe material has been widely used in many applications including catalysis, antibacterial agents, civil as nano-paint (self-cleaning), and especially photocatalysis^5–8^. However, the relatively wide bandgap of TiO_2_ (3.2 eV) limits its photocatalytic activity to harmful UV light i.e. just 5% of the solar energy. Further, it suffers from a low quantum efficiency resulting from the rather fast recombination of electron–hole pairs. To overcome these limitations, various innovative strategies have been developed to improve the photocatalytic properties of TiO_2_. The heterojunctions with other semiconductors, dye sensitization and metal ion implantation, noble metal deposition, elemental doping, and inorganic acids modification are some of these strategies^9–13^. Modification of TiO_2_ with ascorbic acid (vitamin C)^14^ and dendrimers^15–17^, are among our ongoing research activities in this area.

Vitamins are of great interest due to their important biochemical functions and biomedical applications. They have been used vastly in different chemical and biochemical processes inspired by their catalytic or regulatory nature to facilitate or control vital chemical reactions in the human body^18^. B vitamins are important cofactors of enzymatic reactions and among the important vitamins of this group^19–21^, pantothenic acid (vitamin B5), as the obligate precursor of coenzyme A (CoA) and the acyl carrier protein^22,23^.

On the other hand, the highly branched three-dimensional structures with a high affinity to encapsulate the transition metals onto the termini of dendritic tethers or at the dendrimer core have created an impressive position for the design of dendritic catalysts^24,25^. The dendritic-modified TiO_2_ photocatalysts display a high-power conversion efficiency and a high absorption coefficient in visible light irradiation^26^.

**Figure 1:**
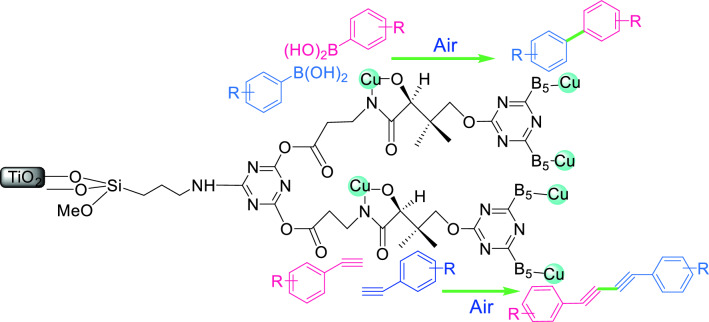
Cu(I) Cu(II) [PTAPA G2-B5]@TiO_2_ catalyzed homocoupling of aryl boronic acid and terminal alkynes.

Our promising results on the visible light photocatalytic activities of vitamins- and dendrimers-modified TiO_2_ photocatalysts in organic transformations^14,15^, was an impetus to design a new dendrimer photocatalyst containing vitamins as branching units. Accordingly, Cu- vitamin B5 was bonded to 2,4,6-trichloro-1,3,5-triazine to produce a bioconjugated dendrimer for modification of nanocrystalline TiO_2_. To the best of our knowledge, this is the first dendrimeric architecture containing branching vitamin units. The dendrimer-based catalyst Cu(I) Cu(II) [Poly triazine pantothenic acid G2-B5]@TiO_2_ (Cu(I) Cu(II) [PTAPA G2-B5]@TiO_2_) induced outstanding visible-light photocatalytic activity for the homocoupling of aryl boronic acid as well as terminal alkynes to biaryl compounds and 1,3-diynes (Glaser-Hay coupling), respectively (Fig. 1).

## Results and discussion

### Synthesis and characterization of the Cu(I) Cu(II) [PTAPA G2-B5] @TiO_2_ catalyst

**Figure 2:**
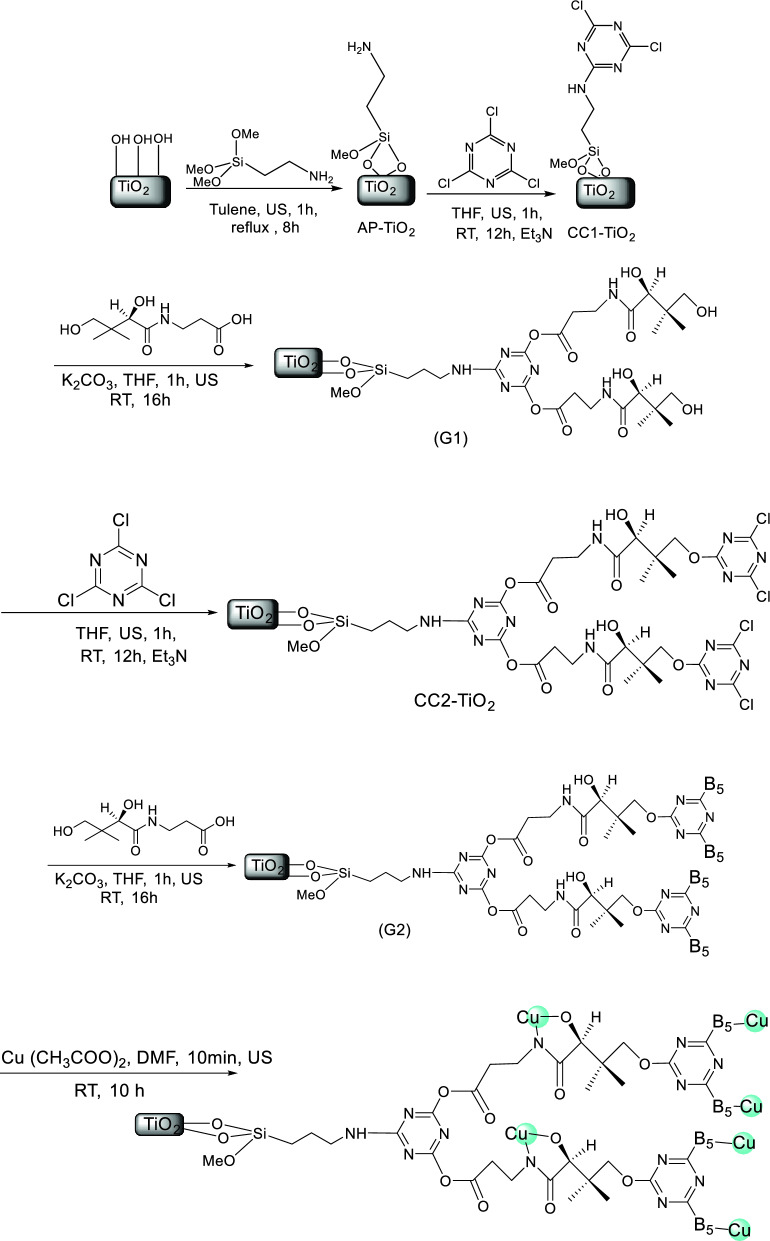
Preparation route for Cu(I) Cu(II) [PTAPA G2-B5] @TiO_2_.

Figure 2 shows the preparation steps of Cu(I) Cu(II) [PTAPA G2-B5] @TiO_2_ dendritic-catalyst. TiO_2_ nanoparticles were synthesized by the polymerizing–complexing (PC) sol–gel method with sizes ranging from 18 to 20 nm^27^, followed by treatment with 3-aminopropyltrimethoxysilane (APTS) to produce amine functionalized TiO_2_ (AP-TiO_2_)^28^. Condensation of cyanuric chloride (CC) with Ap-TiO_2_ at room temperature yielded the CC1-TiO_2_, which upon reaction with pantothenic acid (vitamin B5), furnished G1 dendrimer. Subsequently, the same procedure was used to increase the dendrimer generation, CC2-TiO_2,_ and G2 dendrimer. Finally, the desired copper-containing catalyst (Cu(I) Cu(II) [PTAPA G2-B5] @TiO_2_) was obtained by incorporating Cu(OAc)_2_ to [PTAPA G2-B_5_] @TiO_2_ under sonication and reflux conditions.

The FT-IR spectrum of AP-TiO_2_ showed broad peaks at 3414 and 1644 cm^−1^, which are attributed to hydroxyl groups and the surface adsorbed water. Also, the bands at 500–750 cm^−1^ correspond to the stretching vibration of the Ti–O groups^29^. A strong peak located at 1121–1146 cm^−1^ is attributed to the Si–O bond (Fig. S1a, ESI)^30^. The peaks at 1579–1617 cm^−1^ (C=N) in the FT-IR spectra of b, d–g (Fig. S1b, S2d–e, S3f, g, ESI) confirm the presence of triazine units on the TiO_2_^31^. Two index peaks at 1638, and 1586 cm^−1^ correspond to carbonyl groups of acid and amid groups, respectively (Fig. S1c, ESI). The remarkable spectral changes in Fig. S1d affirm the formation of the TA-B_5_ ligand. The emergence of a new band at 1722 cm^−1^ with some shifting indicates the formation of ester groups conjugated with cyanuric rings. The formation of Cu–N and Cu–O bonds resulting from the complexation of Cu with [PTAPA G2-B5] coated TiO_2_ nanoparticles were evidenced by the bands that appeared at 509 and 682 cm^-1^ respectively, in the FT-IR spectra depicted in Fig. S1g^32,33^.

**Figure 3:**
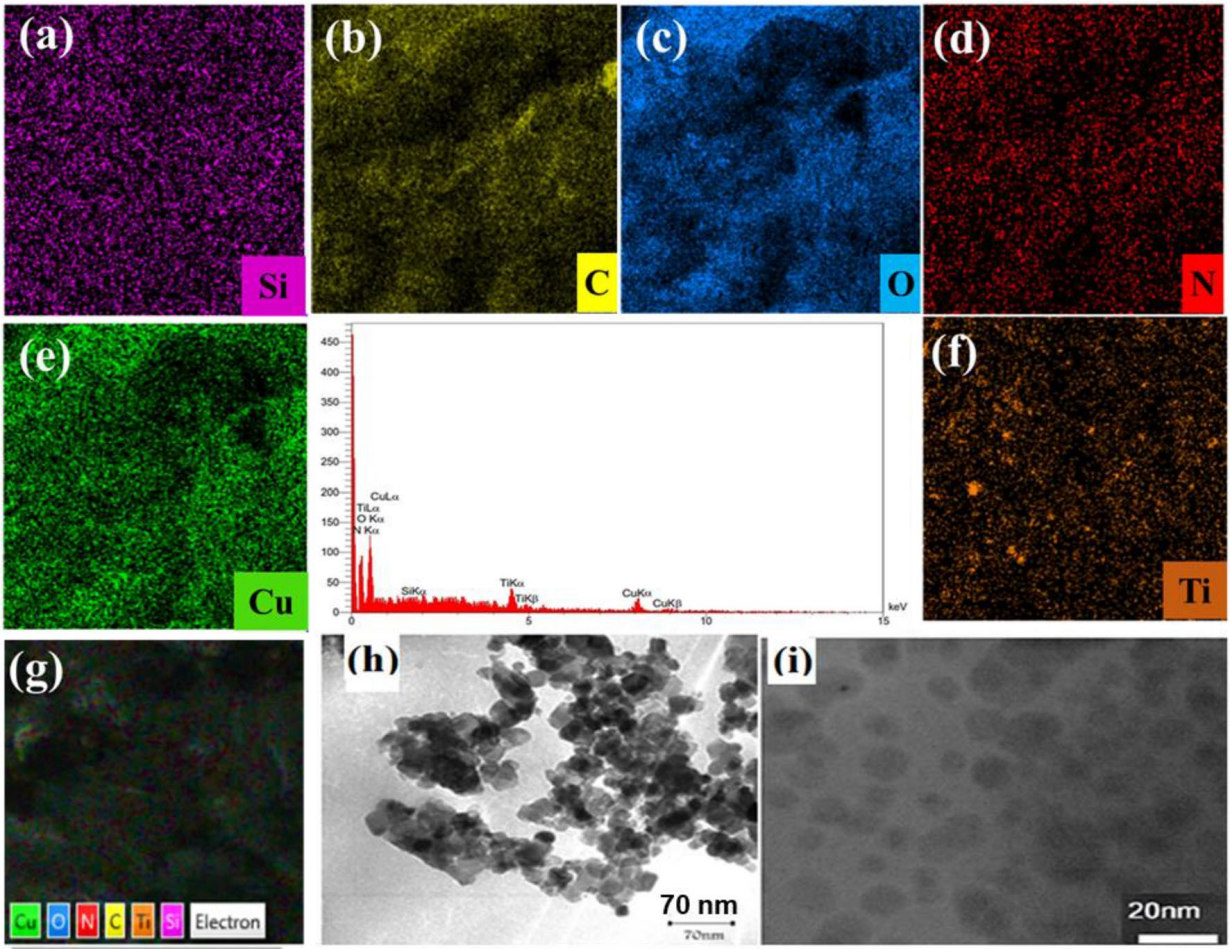
Elemental mapping images (**a–g**) with EDX analysis by SEM, and TEM images of (**h**) pristine TiO_2_, (**i**) as-synthesized dendritic Cu(I) Cu(II) [PTAPA G2-B5] @TiO_2_.

Elemental mapping images (a–g) and EDX analysis by SEM as well as TEM images (h, i) of (Cu(I) Cu(II) [PTAPA G2-B5] @TiO_2_) nanohybrid are shown in Fig. 3 which confirmed the presence of Cu, Ti, Si, N, O and C in the nanosphere heterostructure. The TEM images of the title catalyst revealed well-separated spherical nanoparticles with sizes ranging from 7 to 10 nm. The precise copper content was found to be 1.27 mmol g^−1^ based on the inductively coupled plasma optical emission spectrometry (ICP-OES).

**Figure 4:**
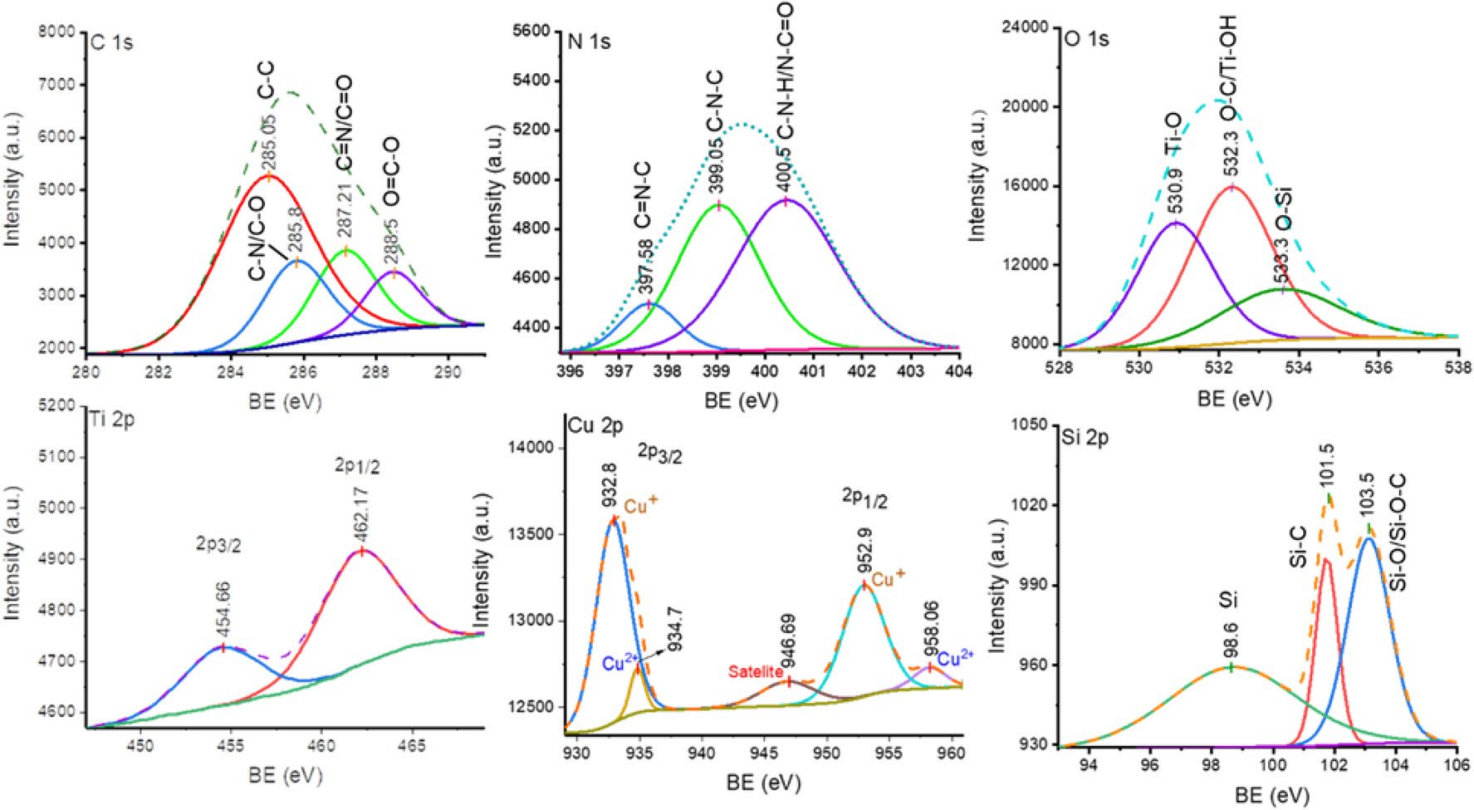
High resolution XPS spectra of composed elements of Cu [PTAPA G2-B5] @TiO_2_.

XPS analysis was carried out to explore the chemical compositions and oxidation states of the composed elements of the title catalyst. The high-resolution XPS spectra of C 1s, N 1s, O 1s, Si 2p, Cu 2p, and Ti 2p are depicted in Fig. 4. The four signals of C 1s located at 285.05, 285.8, 287.21, and 288.5 eV correspond to C–C, C–O/C–N, C=N/C=O, and O=C–O bonds, respectively^34^. The N 1s spectrum was fitted into three peaks centered at 397.58, 399.05, and 400.5 eV, assigned to the sp^2^ hybridized nitrogen (C=N–C), C–N–C, and C–N–H/N–C=O, respectively^35^. The O 1s spectra revealed three peaks at 530.9, 532.3, and 533.3 eV, attributed to Ti–O, C–O/Ti–OH, and O–Si bonds, respectively^36^. In the high-resolution spectra of Cu 2p, two small peaks were observed at 934.7 and 958.19 eV corresponding to 2p_3/2_ and 2p_1/2_ of Cu^2+^. Further, two intense signals located at 932.8 (2p_3/2_) and 952.9 eV (2p_1/2_) testify to the main contribution of Cu(I) oxidation state featuring that the significant reduction of Cu(II) to Cu(I) occurred during the complexation of Cu(II) with dendrimer^37^. Ti 2p_3/2_ and Ti 2p_1/2_ of TiO_2_ also appeared at the binding energies of 454.66 and 462.17 eV at Ti 2p spectra^38^. Further, deconvoluted Si 2p spectra showed two main components at 101.5 eV and 103.15 eV corresponding to Si–C and Si–O/Si–O–C bonds, respectively^39^.

The thermal stability of [PTAPA G1-B5] and nanodendritic catalyst were studied by thermogravimetric analysis. The thermal decomposition curve of the nanodendritic catalyst showed a sequence of two decomposition steps, given in Fig. S2. The first weight loss up to 136 °C attributed to the dehydration of the samples and the second one in the range of 400–423 °C corresponds to the decomposition of organic parts (Fig. S2, ESI).

### Optical properties

**Figure 5:**
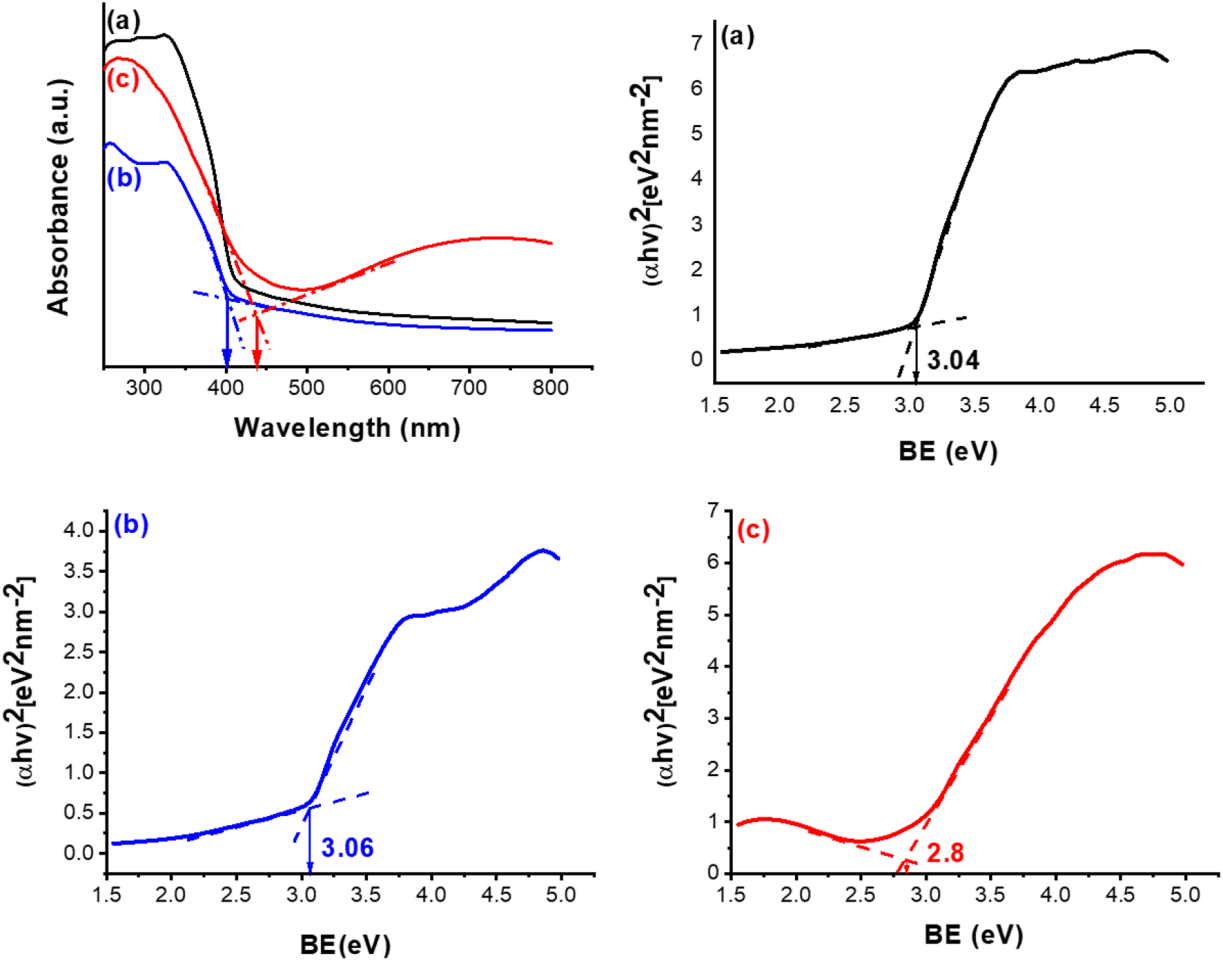
Diffuse reflectance UV–Vis spectra and tauc plots of (**a**) [PTAPA G1-B5] @TiO_2_, (**b**) [PTAPA G2-B5] @TiO_2_ and (**c**) Cu(I) Cu(II) [PTAPA G2-B5] @TiO_2_ nanodendritic catalyst.

The diffuse reflectance UV–Vis spectra (DRS) of [PTAPA G1-B5]@TiO_2_, [PTAPA G2-B5] @TiO_2,_ and the final Cu-containing nanodendritic catalyst (Cu(I) Cu(II) [PTAPA G2-B5] @TiO_2_) were recorded to assess the visible light absorption ability and band gap value (Fig. 5). Comparing the DRS of the bare TiO_2_ possesses an absorbance threshold at less than 400 nm with a band gap of 3.2 eV^40^, our results in this work clearly show the modification roles of the bioconjugated dendrimer as well as Cu complexation on the optical properties of TiO_2_. The absorbance threshold of TiO_2_ shifted to 400 and 440 nm after functionalization with bioconjugated dendrimer and Cu(I) Cu(II) complexation with vitamin B5, respectively (Fig. 5 DRS a–c). As determined by tauc plots^41^, the band gaps reduced to 3.0 and 2.8 eV respectively (Fig. 5, tauc plots a–c). The more important result is that the incorporation of copper increased significantly the amount of absorption of the final nanocomposite (Fig. 5 DRS c) in the wide range of visible regions (500–800 nm).

**Figure 6:**
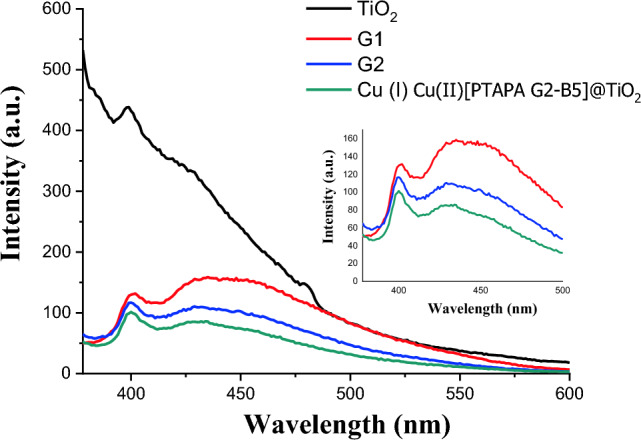
PL spectra of TiO_2_, [PTAPA G1-B5] @TiO_2_ (G1), [PTAPA G2-B5]@TiO_2_ (G2), Cu(I) Cu(II) [PTAPA G2-B5]@TiO_2_ excited at 355 nm.

The possibility of photoinduced electron transfer (PET) between TiO_2_ and denderimer in the resulting hybrids was assessed by PL spectroscopy. The significant decrease in the PL intensity of TiO_2_ after functionalization with PTAPA G1-B5, PTAPA G2-B5, and Cu(I) Cu(II) [PTAPA G2-B5] shows an efficient separation of the carriers resulting from charge transfer between TiO_2_ and dendrimer parts (Fig. 6). The superiority of G2 over G1 can be assigned to the more extensive π-conjugated bonds making it more efficient for both visible light absorption and charge separation. Based on the DRS and PL results, modification of the photoelectronic properties of TiO_2_ seems obvious, which is expected to improve its photocatalytic activity.

### Catalytic activities

The catalytic activity of the as-prepared catalyst was assessed in the homocoupling of aryl boronic acids as well as terminal alkynes to biaryl compounds and 1,3-diynes, respectively. Initially, the reaction conditions such as solvent type and amount, catalyst loading, and temperature under the fluorescent lamp (room light lamps) were optimized using phenylboronic acid as a model substrate (Fig. S3, ESI). The homocoupling of phenylboronic acid (0.125 mmol) reached the highest performance using 6 mol% Cu (I) Cu(II) [PTAPA G2-B5] @TiO_2_ nanodendritic catalyst in 0.1 mL MeOH at 50 °C within 1h under visible light irradiation.

When the catalytic potential of the title catalyst was examined in the homocoupling of phenylacetylenes new optimization conditions were needed. According to the results presented in Fig. S4, for efficient homocoupling of 0.125 mmol phenylacetylene, the reaction needs 5 mol% (0.006 mmol) catalyst and 0.1 ml DMF containing 0.125 mmol Et_3_N and should be heated up to 100 °C (Fig. S4i–vi).

With the optimal reaction conditions in hand, the generality of the method was evaluated using phenylboronic acid and phenylacetylene derivatives. According to the results presented in Table 1, biaryls were generally produced in good to high yields within 2 h, although molecules bearing electron-deficient groups (entries 5, 6), as well as heteroaryl derivatives (entry 7), exhibited less activity. The homocoupling of phenylacetylenes (entries 8, 9) took about 3 h and the pertinent 1,3-diynes produced in moderate to high yields (45–85%), nevertheless, alkyl counterparts were actually inactive under these conditions (entries 10, 11) even after 12 h.

**Table 1:** The C–C homocoupling of different substrates for the synthesis of biaryl and 1,3-diynes derivatives catalyzed by Cu(I) Cu(II) [PTAPA G2-B5] @TiO_2_.


Entry	Substrate	Product^c^	Time (h)	Conversion (%)	Yield (%)^d^
1^a^			1	100	95
2			2	92	85
3			2	100	93
4			1.5	98	89
5			2	65	50
6			1.5	72	65
7			1.5	65	55
8^b^			3	90	85
9			3	57	45
10			12	10	Trace
11			12	10	Trace

^a^The molar ratio of arylboronic acids/ catalyst was 0.125:0.0076. The reactions were run under visible light (room light lamps, 400–650 nm, 40 W) in the air at 50 °C in 0.1 mL MeOH.

^b^The molar ratio of phenylacetylenes/ Et_3_N/ catalyst was 0.125:0.125:0.006. The reactions were run at 100 °C in DMF (0.1 mL) under visible light (room light lamps, 400–650 nm, 40 W).

^c^The products were identified by NMR spectroscopy.

^d^Yields of isolated products.

To show the photocatalytic superiority of the as-prepared Cu(I) Cu(II) [PTAPA G2-B5]@TiO_2_ composite some control experiments for coupling of phenylboronic acid (Table 1, entry 1) and phenylacetylene (Table 1, entry 8) in the presence of the relevant catalysts were performed (Table 2). Under optimized conditions, the bare TiO_2_ was incompetent as a catalyst for homocoupling reaction, and Cu(OAc)_2_ exhibited low to moderate activity to produce 35 and 45% yield of the pertinent biaryl and 1,3-diyne respectively (Table 2, entry 1). Replacement of G2 dendrimer with G1 in the catalyst reduced the homocoupling products to 45 and 40%, respectively (Table 2, entry 3). Given the same effect of the G1 and G2 dendrimers on the band gap of TiO_2_ (Fig. 3a, b), the promoting effect of growing dendritic branches on the photocatalytic activity caused by more effective carriers’ separation resulting from a more extensive system of conjugated π bonds as evidenced by PL spectra (Fig. 6). We also replaced the TiO_2_ core in the Cu(I) Cu(II) [PTAPA G2-B5] @TiO_2_ with MoO_3_ and silica-coated γ–Fe_2_O_3_ nanoparticles which resulted in a significant reduction in the homocoupling performance (25 and 15% for biphenyl and 1,4-diphenylbuta-1,3-diyne, respectively) (Table 2, entries 6, 8). Thus, the presence of TiO_2_ core is inevitable for the photocatalytic activity of the as-prepared (Cu(I) Cu(II) [PTAPA G2-B5] @TiO_2_ nanocomposite.

**Table 2:** The comparison of catalytic activity of Cu(I) Cu(II) [PTAPA G2-B5] @ TiO_2_ nanodendritic catalyst with other nanocomposites for homo-coupling reactions.

Entry	Catalyst	Biphenyl %^a^	1,4-Diphenylbuta-1,3-diyne %^b^
1	Cu(OAc)_2_	35	45
2	TiO_2_	0	10
3	Cu(I) Cu(II) [PTAPA G1-B5] @TiO_2_	45	40
4	Cu(I) Cu(II) [PTAPA G2-B5]@TiO_2_	100	90
5	MoO_3_	10	20
6	Cu [PTAPA G2-B5] @ MoO_3_	25	25
7	SMNP	0	10
8	Cu [PTAPA G2-B5]@ SMNP	15	15

^a^Phenylboronic acid conversion.

^b^Phenylacetylene conversion under optimized conditions.

**Figure 7:**
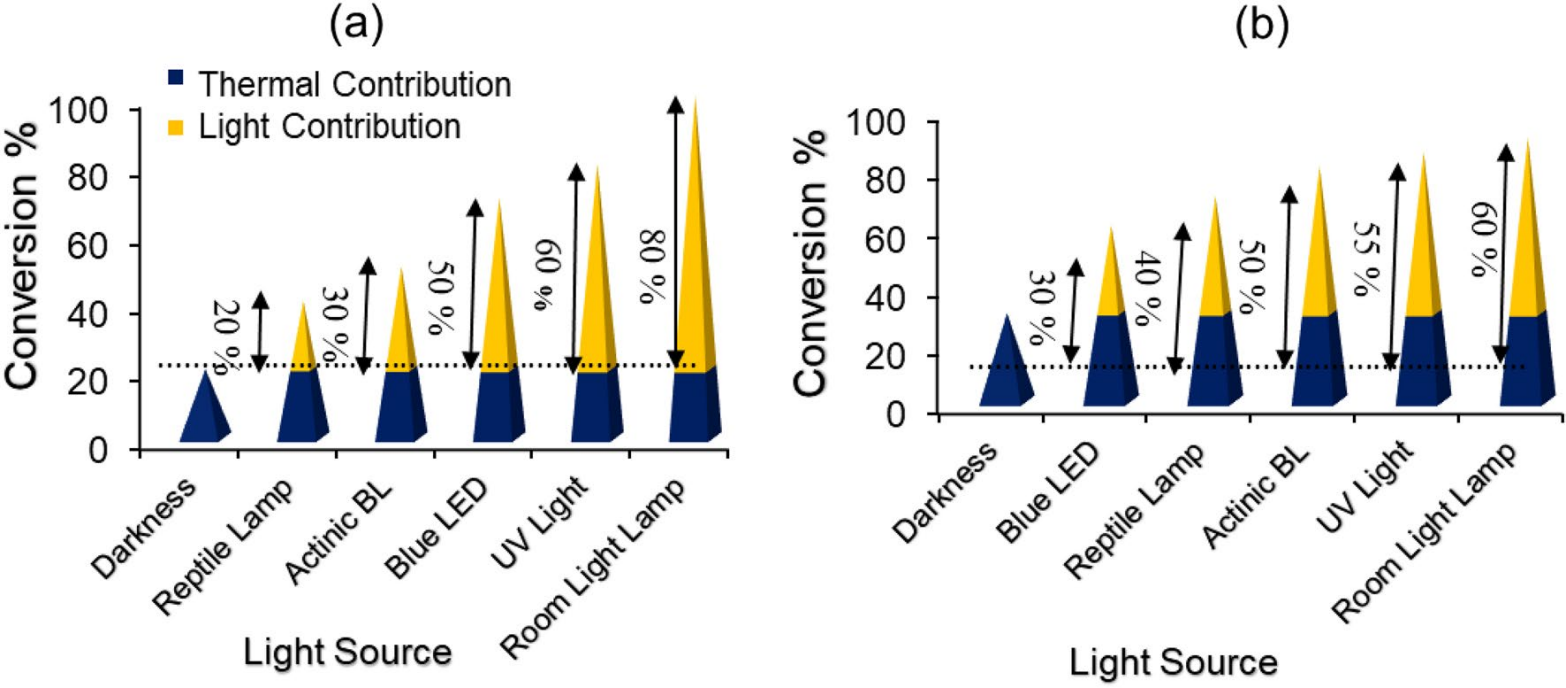
Dependence of the catalytic activity of the Cu(I) Cu(II) [PTAPA G2-B5] @ TiO_2_ for the homocoupling reactions of (**a**) phenylboronic acid and (**b**) phenylacetylene on the irradiation wavelength. The numbers with percentages show the contribution of the light irradiation effect. (**a**) Reaction condition: 0.125:0.0076 mmolar ratio for phenylboronic acid: catalyst, MeOH (0.1 mL) at 50 °C after 1h. (**b**) Reaction condition: 0.125:0.125:0.006 molar ratio for phenylacethylene:Et_3_N:catalyst, DMF (0.1 mL) at 100 °C after 3h.

The light dependent catalytic activity of the Cu(I) Cu(II) [PTAPA G2-B5]@TiO_2_ was also assessed under various light sources such as Reptile lamp, LT NARVA (18 W, full range visible light + 4% UV), Actinic BL TL-D Philips (15 W, *λ* = 366–400 nm), and blue LED, AC86, Z.F.R (12 W, *λ*max = 505 nm), UV light (λ = 200–290 nm, 15 W), room light lamps (Fluorescent lamp, λ = 400–650 nm, 40 W). The light contributions of homocoupling reaction of phenylboronic acid and phenylacetylene under the aforementioned lamps were depicted in Fig. 7a, b. In both reactions, the room light lamp exhibited the greatest irradiation contribution to the overall conversion rate. Different light contributions under light sources with different emission wavelength ranges clearly show that the reactions are light dependent.

### Mechanism study

**Figure 8:**
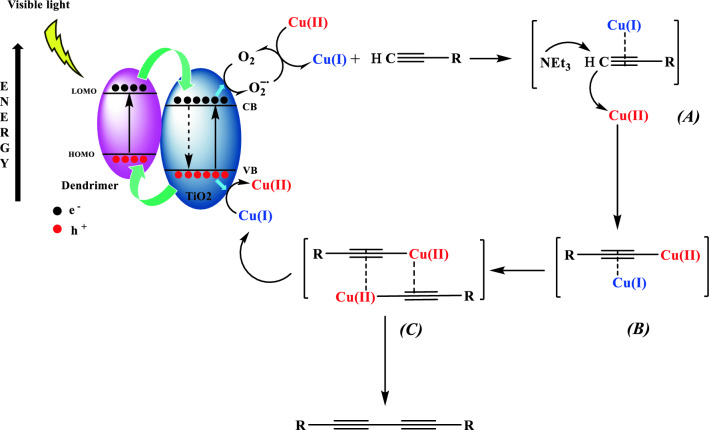
Proposed mechanism for homo coupling reaction of phenylacetylene.

Given the results of the photocatalytic assessment mentioned in the previous section and previous studies^42^, a photocatalytic process is definite for Cu(I) Cu(II) [PTAPA G2-B5] @ TiO_2_. Irradiating the catalyst excites an electron from the HOMO of dendrimer to its LUMO leaving holes (h+) in the HOMO, forming electron–hole pairs (Fig. 8). For the CB of TiO_2_ matched the LUMO level of dendrimer well for the charge transfer, this excited state dendrimer species can be converted to a semi-oxidized radical cation (D^•+^) by the injection of an electron into the CB of TiO_2_. In line with this hypothesis, G2 with a more extensive system of conjugated π bonds than G1 is expected to absorb light more efficiently in the visible range and also facilitate electron transfer providing more effective charge separation. Those electrons of the TiO_2_ conduction band were captured by the O_2_ pre-adsorbed on the TiO_2_ surface to form a superoxide anion radical (O^•−2^) capable of reducing Cu(II) to Cu(I). At the end of the reaction, on the other hand, the Cu(I) can be reoxidized to Cu(II) by photogenerated holes on the VB of TiO_2_ to complete the photocatalytic cycle. As the redox reaction goes on, the number of electrons injected and the holes produced in TiO_2_ gradually increases, yielding more reactive radical species on the surface of TiO_2_, thus increasing the photocatalytic activity of the composite photocatalyst. To prove this claim, the homocoupling reaction of phenylacetylene (and phenylboronic acid) in the presence of benzoquinone as superoxide radical scavenger and ammonium oxalate (AO) and formic acid (FA) as hole scavengers as well as 2,6-Di-tert-butyl-4-methylphenol (BHT) and TEMPO as common radical scavengers under light irradiation were performed (Fig. S5). The conversion of phenylacetylene reduced to 34, 22, 15, 35 and 31% respectively (28, 17,10, 20, and 25 for phenylboronic acid respectively), much close to the results obtained in darkness attributed to the contribution of thermal effect.

Based on the above results and previous reports, a radical mechanism relying on the photogenerated e− and h+ assisted by Cu(I)–Cu(II) synergistic cooperation is proposed^43^. As displayed in Fig. 8, the superoxide radicals produced by photogenerated electrons on CB of TiO_2_, reduce Cu(II) to Cu(I) followed by attachment to terminal alkyne molecules to generate a coordination adduct intermediate (A). By this step, the inactive C−H bond could be activated (*A*) to be deprotonated by NEt_3_ forming C−Cu(II) bond to generate intermediate *B*. The next step is the dimerization of B to generate intermediate *C*. Finally, C is involved in the electron transfer and C−C bond formation step to produce a homo-coupled product and regenerate the reduced Cu(I) species to drive a new cycle by cooperation of Cu(II) (Fig. 8). By inspection of the aforementioned mechanism steps, the catalytic role of Cu(I) seems more dominant than Cu(II) matching well with the high activity of the title catalyst possessing mainly Cu(I) evidenced by XPS analysis (Fig. 4). The Cu(I) coordinates with alkyne molecules to activate C–H bond followed by a rapid electron-transfer step from Cu(II) leading to the homocoupling product. The overall reaction is driven by involving Cu(I)–Cu(II) synergistic cooperation. (See proposed mechanism as Fig. S6 for homo coupling reaction of phenylboronic acid in SI)^44^.

Therefore, by coupling the TiO_2_ semiconductor with a dendrimer, a greater photocatalytic performance under visible light irradiation can be achieved as the dendrimer increase the efficiency of sunlight utilization in the visible light range and lower the rate of electron–hole pair recombination in TiO_2_.

### Recycling test

The promising results for the catalytic activity of Cu(I)Cu(II)[PTAPAG2-B5]@TiO_2_ nanocomposite encouraged us to assess its reusability in sequential reactions. As can be seen in Fig. S7 the catalyst preserved its activity during five runs for homocoupling reactions of both phenylboronic acid and phenylacetylene under optimized reaction conditions. Further, the FT-IR spectra of the reused nanocatalyst depicted in Figs. S8 and S9 revealed that the catalyst maintained its structural integrity during the reaction. However, to confirm the bonding between the catalyst’s components, a leaching experiment (hot filtration test) was operated for the homocoupling reaction of phenylboronic acid under optimized conditions (Table 1, entry 1). The catalyst was quickly removed after 15 min in which the conversion of substrate reached 40%, and the filtrate was allowed to stir at 50 °C for a further 45 min. No progress in the reaction was detected confirming that the catalyst’s components are held tightly and the Cu(I)Cu(II)[PTAPAG2-B5]@TiO_2_ did act as a heterogeneous catalyst (Figure S10, SI). The ICP-OES analysis of both the filtrate and the used catalyst also excluded any leaking of Cu providing further evidence for the heterogeneous nature of the title photocatalytic system. No Cu was detected in the filtrate and the precise Cu content of the used catalyst was 1.25 mmol g^−1^ corresponding to a 1.6% loss of Cu in comparison with the fresh catalyst (1.27 mmol g^−1^) that is ignorable in the range of analysis error.

## Conclusion

In conclusion, combining 2,4,6-trichloro-1,3,5-triazine with vitamin B5 followed by complexation with copper rendered a new bioconjugated dendrimer to improve effectively the visible-light photocatalytic activity of TiO_2_ nanoparticles. The coexistence of Cu(II)/Cu(I) oxidation states with a predominant contribution of Cu(I) was uncovered by XPS analysis. The DRS and PL spectra showed that the copper-containing dendrimer reduced the band gap value as well as increased the charge separation resulting in the promotion of the visible-light photocatalytic activity of TiO_2_. Moreover, the incorporation of copper into the bioconjugated dendrimer increased significantly the absorption amount of the final nanocomposite in the wide range of visible regions (500–800 nm). The heterogeneous aerobic homocoupling of phenylboronic acid and phenylacetylene was successfully driven by the as-prepared bio-nanocatalyst under the visible light and the catalyst retained its activity and structural integrity after several recyclings. The reactions showed light dependency and the room light lamp exhibited the greatest irradiation contribution to the overall conversion rate. The improved photoactivity of the as-prepared nanohybrid predominantly benefits from the synergistic effects of Cu(I) Cu(II) [PTAPA G2-B5] and TiO_2_ nanoparticles relying on the extensive conjugated π bonds of dendrimer in a heterojunction structure. A radical mechanism based on the photogenerated e− and h+ and involving the Cu(I)–Cu(II) synergistic cooperation was proposed. The present system that employs an air-stable, very active, robust, and recyclable photocatalyst under a visible light source qualifies the significant conditions for implementation in the industry. This strategy will open up a new outlook for better use of semiconducting materials in photochemical applications and sequential organic transformations.

## Experimental

Note: General remarks and preparation steps of Cu(I)Cu(II) [PTAPA G2-B5] @TiO_2_ catalyst are given in SI.

### General experimental procedure for aerobic homocoupling reaction of arylboronic acids in the presence of Cu(I) Cu(II) [PTAPA G2-B_5_] @TiO_2_

Aryl boronic acid (0.125 mmol), Cu(I) Cu(II) [PTAPA G2-B_5_] @TiO_2_ (0.0076 mmol, 0.006 g, 6 mol%), and methanol (0.1 mL) were placed in an open tube (10 cm height, 1.5 cm diameter, and 14 span), and stirred at 50 °C in the air, and room light lamps (λ = 400–650 nm, P = 40 W) for the needed time. The reaction progress was monitored by TLC. At the end of the reaction, the catalyst was separated by centrifuging, and then the product (liquid phase) was extracted by plate chromatography eluted with n-hexane/EtOAc (10/1).

### General experimental procedure for homocoupling reaction of acetylenes catalyzed by Cu(I) Cu(II) [PTAPA G2-B_5_]@TiO_2_

To a mixture of 0.006 mmol (0.005 g, 5 mol%) catalyst, and 0.1 mL DMF in a close tube (10 cm height, 1.5 cm diameter, and 14 span) were added 0.125 mmol Et_3_N and phenylacetylenes, then stirred at 100 °C under room light lamps (λ = 400–650 nm, P = 40 W) for the required time. The reaction progress was monitored by TLC. At the end of the reaction, the reaction mixture was cooled to room temperature, the product was extracted by decantation EtOAc (3 $\times$ 0.2 mL). The solvent was removed in vacuum and obtained pure products.

### Reusability of the catalyst

To a mixture of phenylboronic acid (0.625 mmol) and (30mg) Cu(I) Cu(II) [PTAPA G2-B_5_] @TiO_2_] was added MeOH (0.5 mL), and stirred in the air at 50 °C for the needed time. At the end of the reaction, after cooling the reaction mixture, the catalyst was extracted by centrifuging and dried in a vacuum oven to be reused for the next runs. The reusability of the catalyst was also investigated in the homocoupling reaction of acetylenes.

### Supplementary Information

Supplementary Information.

## Data Availability

All data generated or analyzed during this study are included in this published article [and its supplementary information files].

## References

[1] Bora LV, Mewada RK (2017). Visible/solar light active photocatalysts for organic effluent treatment: Fundamentals, mechanisms and parametric review. Renew. Sustain. Energy Rev..

[2] Pandey S, Mandari KK, Kim J, Kang M, Fosso-Kankeu E (2020). Recent advancement in visible-light-responsive photocatalysts in heterogeneous photocatalytic water treatment technology. Photocatal. Adv. Oxid. Process. Wastewater Treat..

[3] Franchi D, Amara Z (2020). Applications of sensitized semiconductors as heterogeneous visible-light photocatalysts in organic synthesis. ACS Sustain. Chem. Eng..

[4] Asadzadeh-Khaneghah S, Habibi-Yangjeh A (2020). g-C_3_N_4_/carbon dot-based nanocomposites serve as efficacious photocatalysts for environmental purification and energy generation: A review. J. Clean. Prod..

[5] Kumar A, Choudhary P, Kumar A, Camargo PHC, Krishnan V (2022). Recent advances in plasmonic photocatalysis based on TiO_2_ and noble metal nanoparticles for energy conversion, environmental remediation, and organic synthesis. Small.

[6] Wu Y (2021). Synthesis of high-performance conjugated microporous polymer/TiO_2_ photocatalytic antibacterial nanocomposites. Mater. Sci. Eng. C.

[7] Abd Aziz, A., Khatun, F., Monir, M. U., Ching, S. L. & Hon, L. K. TiO_2_: A semiconductor photocatalyst. In *Titanium Dioxide-Advances and Applications* (IntechOpen, 2021).

[8] Haider AJ, Jameel ZN, Al-Hussaini IHM (2019). Review on: Titanium dioxide applications. Energy Proc..

[9] Cheng H, Xu W (2019). Recent advances in modified TiO_2_ for photo-induced organic synthesis. Org. Biomol. Chem..

[10] Ansari SA, Khan MM, Ansari MO, Cho MH (2016). Nitrogen-doped titanium dioxide (N-doped TiO_2_) for visible light photocatalysis. New J. Chem..

[11] Basavarajappa PS (2020). Recent progress in metal-doped TiO_2_, non-metal doped/codoped TiO_2_ and TiO_2_ nanostructured hybrids for enhanced photocatalysis. Int. J. Hydrogen Energy.

[12] Patil SB (2019). Recent advances in non-metals-doped TiO_2_ nanostructured photocatalysts for visible-light driven hydrogen production, CO_2_ reduction and air purification. Int. J. Hydrogen Energy.

[13] Chen J, Qiu F, Xu W, Cao S, Zhu H (2015). Recent progress in enhancing photocatalytic efficiency of TiO_2_-based materials. Appl. Catal. A Gen..

[14] Pourmorteza N, Jafarpour M, Feizpour F, Rezaeifard A (2022). Cu (ii)–vitamin C-complex catalyzed photo-induced homocoupling reaction of aryl boronic acid in base-free and visible light conditions. RSC Adv..

[15] Eskandari A, Jafarpour M, Rezaeifard A, Salimi M (2018). A dendritic TiO_2_–Co (ii) nanocomposite based on the melamine catalyzed one-pot aerobic photocatalytic synthesis of benzimidazoles. New J. Chem..

[16] Jung J-J, Jang J-W, Park J-W (2016). Effect of generation growth on photocatalytic activity of nano TiO_2_-magnetic cored dendrimers. J. Ind. Eng. Chem..

[17] Maleki A, Hayati B, Najafi F, Gharibi F, Joo SW (2016). Heavy metal adsorption from industrial wastewater by PAMAM/TiO_2_ nanohybrid: Preparation, characterization and adsorption studies. J. Mol. Liq..

[18] Tanz, R. D. & Urquilla, P. R. Cardiac glycosides. In *Modern Pharmacology* (Craig, C.R., Stitzel, R.E. eds.). 269–281 (1982).

[19] Acevedo-Rocha CG, Gronenberg LS, Mack M, Commichau FM, Genee HJ (2019). Microbial cell factories for the sustainable manufacturing of B vitamins. Curr. Opin. Biotechnol..

[20] Monteverde DR, Gómez-Consarnau L, Suffridge C, Sañudo-Wilhelmy SA (2017). Life’s utilization of B vitamins on early Earth. Geobiology.

[21] Baj, T. & Sieniawska, E. Vitamins. Chap. 13. In: *Pharmacognosy: Fundamentals, Applications and Strategies* (2017).

[22] Douglas AE (2017). The B vitamin nutrition of insects: The contributions of diet, microbiome and horizontally acquired genes. Curr. Opin. Insect Sci..

[23] Xu J (2020). Cerebral deficiency of vitamin B5 (d-pantothenic acid; pantothenate) as a potentially-reversible cause of neurodegeneration and dementia in sporadic Alzheimer’s disease. Biochem. Biophys. Res. Commun..

[24] Astruc D, Boisselier E, Ornelas C (2010). Dendrimers designed for functions: from physical, photophysical, and supramolecular properties to applications in sensing, catalysis, molecular electronics, photonics, and nanomedicine. Chem. Rev..

[25] Pan S, Yan S, Osako T, Uozumi Y (2017). Batch and continuous-flow Huisgen 1, 3-dipolar cycloadditions with an amphiphilic resin-supported triazine-based polyethyleneamine dendrimer copper catalyst. ACS Sustain. Chem. Eng..

[26] Rajakumar P (2010). Synthesis of triazole dendrimers with a dimethyl isophthalate surface group and their application to dye-sensitized solar cells. New J. Chem..

[27] Jafarpour M, Rezaeifard A, Ghahramaninezhad M, Tabibi T (2013). Reusable α-MoO_3_ nanobelts catalyzes the green and heterogeneous condensation of 1, 2-diamines with carbonyl compounds. New J. Chem..

[28] Karimi B, Safari AA (2008). One-pot synthesis of α-aminonitriles using a highly efficient and recyclable silica-based scandium (III) interphase catalyst. J. Organomet. Chem..

[29] Pourmorteza N, Jafarpour M, Feizpour F, Rezaeifard A (2020). Cu (ii) vitamin C tunes photocatalytic activity of TiO_2_ nanoparticles for visible light-driven aerobic oxidation of benzylic alcohols. RSC Adv..

[30] Eskandari A, Jafarpour M, Rezaeifard A, Salimi M (2019). Supramolecular photocatalyst of palladium (II) encapsulated within dendrimer on TiO_2_ nanoparticles for photo-induced Suzuki-Miyaura and Sonogashira cross-coupling reactions. Appl. Organomet. Chem..

[31] Hasanpour B, Jafarpour M, Feizpour F, Rezaeifard A (2021). Copper (II)-ethanolamine triazine complex on chitosan-functionalized nanomaghemite for catalytic aerobic oxidation of benzylic alcohols. Catal. Lett..

[32] Ansari Z, Saha A, Singha SS, Sen K (2018). Phytomediated generation of Ag, CuO and Ag–Cu nanoparticles for dimethoate sensing. J. Photochem. Photobiol. A Chem..

[33] Batool SS (2019). Synthesis and structural characterization of a monomeric mixed ligand copper (II) complex involving *N, N, N′, N*′-tetramethylethylenediamine and mefenamate. J. Struct. Chem..

[34] Dang VD, Ganganboina AB, Doong RA (2020). Bipyridine- and copper-functionalized N-doped carbon dots for fluorescence turn off-on detection of ciprofloxacin. ACS Appl. Mater. Interfaces.

[35] Fageria P (2016). Synthesis of monometallic (Au and Pd) and bimetallic (AuPd) nanoparticles using carbon nitride (C_3_N_4_) quantum dots via the photochemical route for nitrophenol reduction. Langmuir.

[36] Amanchi SR, Ashok Kumar KV, Lakshminarayana B, Satyanarayana G, Subrahmanyam C (2019). Photocatalytic hydrogenation of nitroarenes: Supporting effect of CoOx on TiO_2_ nanoparticles. New J. Chem..

[37] Ivanova TM (2020). XPS detection of unusual Cu(II) to Cu(I) transition on the surface of complexes with redox-active ligands. J. Electron Spectrosc. Relat. Phenom..

[38] Herman GS, Gao Y, Tran TT, Osterwalder J (2000). X-ray photoelectron diffraction study of an anatase thin film: TiO_2_(001). Surf. Sci..

[39] Kaur A, Chahal P, Hogan T (2016). Selective fabrication of SiC/Si diodes by excimer laser under ambient conditions. IEEE Electron. Dev. Lett..

[40] Ou NQ (2019). Facet-dependent interfacial charge transfer in TiO_2_/nitrogen-doped graphene quantum dots heterojunctions for visible-light driven photocatalysis. Catalysis.

[41] Makuła P, Pacia M, Macyk W (2018). How to correctly determine the band gap energy of modified semiconductor photocatalysts based on UV–Vis spectra. J. Phys. Chem. Lett..

[42] Mansoob Khan, M., Pradhan, D. & Sohn, Y. (eds.) *Nanocomposites for Visible Light-Induced Photocatalysis*10.1007/978-3-319-62446-4.

[43] Bai R (2014). Cu(II)–Cu(I) synergistic cooperation to lead the alkyne C–H activation. J. Am. Chem. Soc..

[44] Cheng G, Luo M (2011). Homocoupling of arylboronic acids catalyzed by CuCl in air at room temperature. Eur. J. Org. Chem..

