# Author Correction: Vitamin B5 copper conjugated triazine dendrimer improved the visible-light photocatalytic activity of TiO_2_ nanoparticles for aerobic homocoupling reactions

**DOI:** 10.1038/s41598-024-54629-1

**Published:** 2024-02-21

**Authors:** Samira Zamenraz, Maasoumeh Jafarpour, Ameneh Eskandari, Abdolreza Rezaeifard

**Affiliations:** Catalysis Research Laboratory, Department of Chemistry, Faculty of Science, University of Birjand, Birjand, 97179-414 Iran

Correction to: *Scientific Reports* 10.1038/s41598-024-52339-2, published online 1 February 2024

In the original version of this Article, the email address of the corresponding author Maasoumeh Jafarpour was incorrect. Correspondence and requests for materials should be addressed to mjafarpour@birjand.ac.ir

The original Article has been corrected.

