# Supplementary material for: Vitamin B5 copper conjugated triazine dendrimer improved the visible-light photocatalytic activity of TiO2 nanoparticles for aerobic homocoupling reactions
Source: Sci Rep. 2024 Feb 1;14:2691. doi: 10.1038/s41598-024-52339-2 (PMC10834398; doi:10.1038/s41598-024-52339-2)
Supplement: Supplementary file 1 — Supplementary Information. [file 41598_2024_52339_MOESM1_ESM.pdf]

Supporting Information

Vitamin B5 Copper (II)-Conjugated Triazine Dendrimer Improved the Visible-Light Photocatalytic Activity of TiO_2 Nanoparticles for Aerobic Homocoupling Reactions

Samira Zamenraz, Maasoumeh Jafarpour*, Ameneh Eskandari, Abdolreza Rezaeifard*

[a] Catalysis Research Laboratory, Department of Chemistry, Faculty of Science, University of Birjand, Birjand, 97179-414 Iran

Experimental

Instrumentation

All chemicals were purchased from Merck and Fluka Chemical Companies. FT-IR spectra were recorded on a JASCO 6300 spectrometer in the range of 400-4000 cm^{-1}. Thermogravimetric analysis (TGA) of powders carried out on NETZSCH TG 209 F1 Iris under N_2 atmosphere flow at a uniform heating rate of 10 ˚$Cmin^{-1}$ in the range of 30-890 ˚C. Transmission electron microscopy (TEM) measurements were obtained by a 906 E instrument (Zeiss, Jean, Germany). Samples for the TEM experiment were prepared by dispersion in ethanol, sonicating for 30 min, and evaporating one drop of solution onto a 200 mesh formbar-coated copper grid. X-ray photoelectron spectroscopy (XPS) measurements were performed using the electron spectrometer SPECS FlexPS. X-ray photoelectron spectroscopy (XPS) was carried out using a non-monochromatized Al Kα source (VG) at 1486 eV under a vacuum (3.1 10^{-9} mbar). Sample preparation: A 1x1 cm tablet of the sample Cu(I)Cu(II)[PTAPAG2-B5]@TiO_2 was prepared using carbon glue. The Cu content of the catalyst was measured by an inductively coupled plasma optical emission spectroscopy (ICP-OES), using a VISTA-PRO ICP analyzer. Sample preparation: a 0.1 g sample was added to an autoclave containing 50 ml concentrated acid mixture (HCl: HNO_3 =3:1) and 50 ml DI water and heated at 200 °C for 15 min. Diffuse reflectance UV-vis spectra were recorded using an Avantes spectrometer (Avaspec-2048-TEC model). EDX spectrum was

recorded by the TESCAN Vega Model instrument. Progresses of the reactions were monitored by TLC using silica-gel SIL G/UV 254 Plates. The light sources are Reptile lamp, LT NARVA (18 W, full range visible light + 4% UV), Actinic BL TL-D Philips (15 W, λ = 366−400 nm), and blue LED, AC86, Z.F.R (12 W, λmax = 505 nm), UV light (λ =200-290 nm, 15 W), room light lamps (Fluorescent lamp, λ = 400-650 nm, P= 40 W).

Fabrication of TiO_2 nanoparticles[1]

To a solution of $TiCl_3$ (0.05 mol) in a mixture of double-distilled water and absolute ethanol (1:1) was added citric acid (0.15 mol) and ethylene glycol (0.15 mol) subsequently. The resulting mixture was dissolved at 45 ˚C under ultrasonic agitation (40 MHz) for 15 min to give a clear violet solution. The solution was refluxed at 120 ˚C for 8 h which turned into a metal- citrate homogeneous complex with a little color change from clear violet to black-violet. After cooling down, in order to bring about the required chemical reactions for the development of polymerization and evaporation of the solvent, the sol was further slowly heated at 90 ˚C for 6 h in an open bath until a beige wet gel was obtained. During continuous heating at this temperature, the polymerization between citric acid, ethylene glycol, and complexes is developed and ultimately sol became more viscous as a wet gel. In the final step of the sol-gel process, the wet gel was fully dried by direct heating on the hot plate at 150 ˚C for 6 h leading to a black powder. Then the calcination was done at 600 ˚C for 3 h with a heating rate of 5 ˚C min^{-1}. Finally, TiO_2 nanoparticles with a white color were obtained.

Preparation of aminopropylated nano titanium dioxide (AP-TiO_2)[2]

For the preparation of AP-TiO_2, the nano-titanium dioxide was fabricated according to the literature procedure.[22] To a 3.0 g TiO_2 nanoparticles in the 50 mL anhydrous toluene was added 8 mL 3-aminopropyltrimethoxysilane (APTS) at room temperature under ultrasonic agitation (40 MHz) for 1 h, then was stirred under reflux conditions for 8 h. Afterward, the reaction mixture was centrifuged and washed with toluene and dried in a vacuum oven at 110 ˚C.

Preparation of triazine-pantothenic acid dendrimer supported on nano titanium dioxide [PTAPA G2-B5]@TiO_2 (G2)

A mixture of AP-TiO_2 (2g, 0.99 mmol/g), cyanuric chloride (CC) (1.85 g, 10 mmol), and triethylamine (Et_3N) (10 mmol, 1.7 mL) in the dry THF (10 mL) was dispersed under ultrasound

agitation (40 MHz) at room temperature for 1 h, then was shaken overnight at room temperature. The solid white material (CC1-TiO_2) was separated by centrifuge, washed with dry THF, and dried in a vacuum oven at 50 ˚C. Then, to a solution of CC1-TiO_2 (1 g) in dry THF (12 mL) was added pantothenic acid (Vitamin B5) (8.11 mmol) and K_2CO_3 (8.11 mmol). The reaction mixture was dispersed under ultrasounic agitation (40 MHz) at room temperature for 1 h, then stirred under room temprature for 16 h. The product first generation (G1) was centrifuged and washed with dry THF, then dried in a vacuum oven at 30 ˚C.

The second-generation [PTAPA G2-B5]@TiO_2 (G2) was prepared following the above procedure for the preparation of G1. A mixture of G1 (1 g) and cyanuric chloride (CC) (1.66 g, 9 mmol) in dry THF (20 mL) was treated at room temperature under ultrasonic agitation (40 MHz) for 1 h. Then, the reaction mixture was shaken at room temperature for 16 h. Afterward, the product was separated by centrifuge, washed with dry THF, and dried in a vacuum oven at 30 ˚C. The resulting solid light yellow material (CC2-TiO_2) was obtained. Then, to a solution of CC2-TiO2 (1 g) in dry THF (20 mL) was added pantothenic acid (9.36 mmol) and K_2CO_3 (9.36 mmol). The reaction mixture was treated at room temperature under ultrasonic agitation for 1 h, then stirred at room temperature for 16 h. The resulting second-generation of nano-titanium dioxide supported dendrimer ([PTAPA G2-B5]@TiO_2 (G2)) was centrifuged and washed with dry THF, then dried in a vacuum oven at 30 ˚C.

1.5. Preparation of Cu(II) [PTAPA G2-B5]@TiO_2

To a mixture of TiO_2 nanoparticles supported dendrimer (G2) (0.5 g) was gradually added $Cu(OAc)_2$ (0.5 mmol) dissolved in DMF over a period of 60 min under ultrasonic agitation (40 MHz) at room temperature. Then, the as-obtained mixture was stirred at room temperature for 10 h. Afterward, the product was centrifuged and washed with ethanol. Finally, Cu(II) [PTAPA G2-B5] @ TiO_2 catalyst was obtained after drying in a vacuum oven at 30 ˚C.

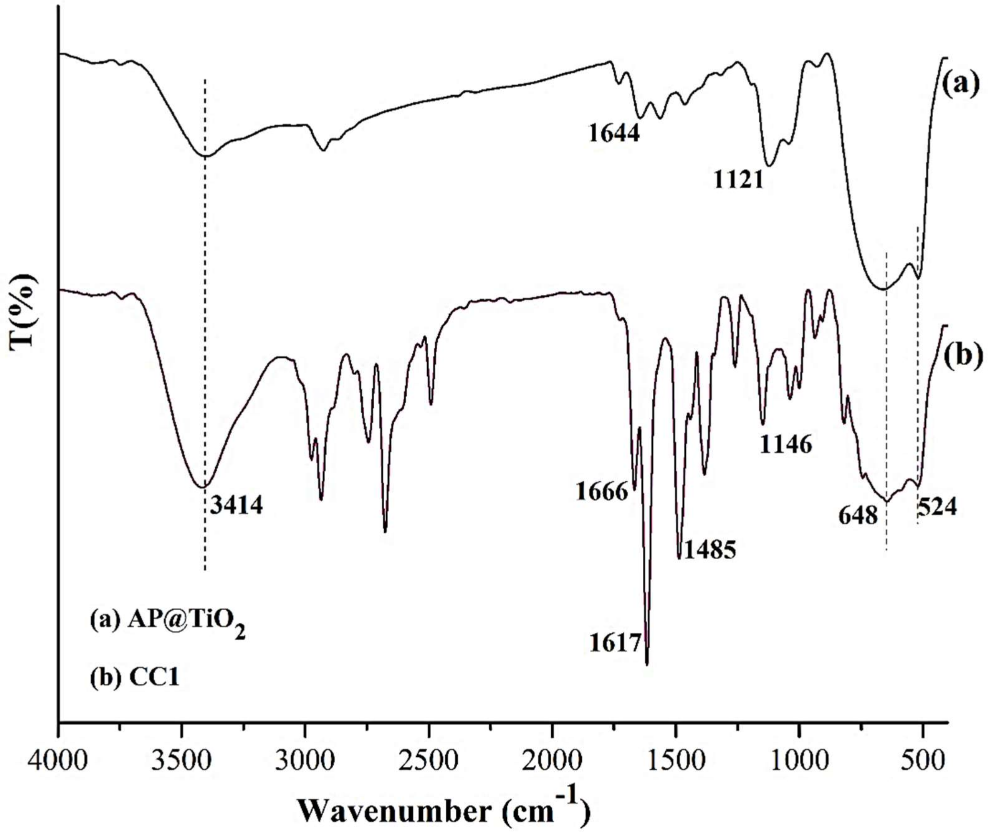

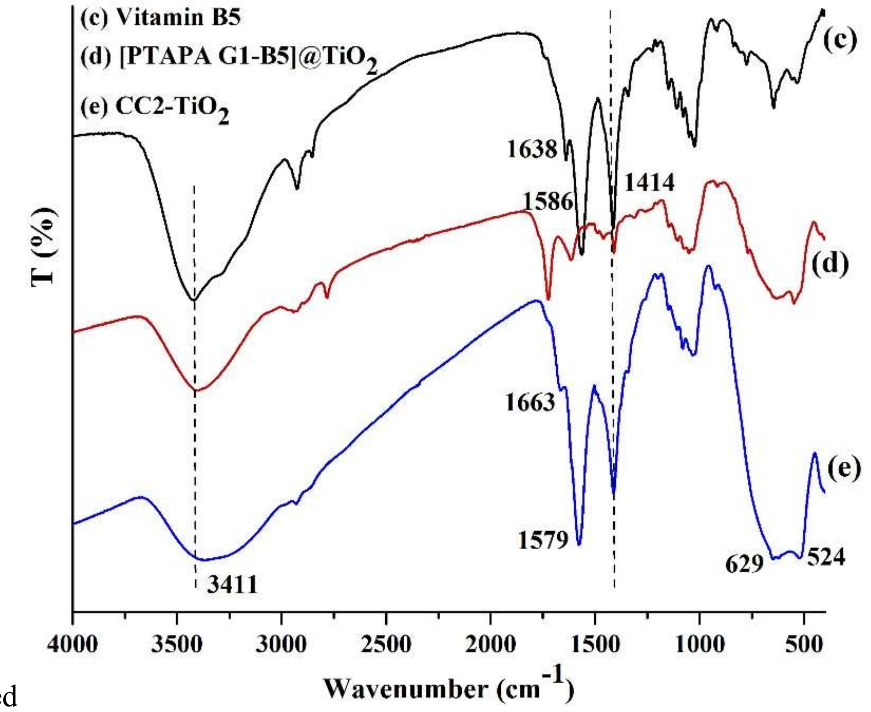

to be continued

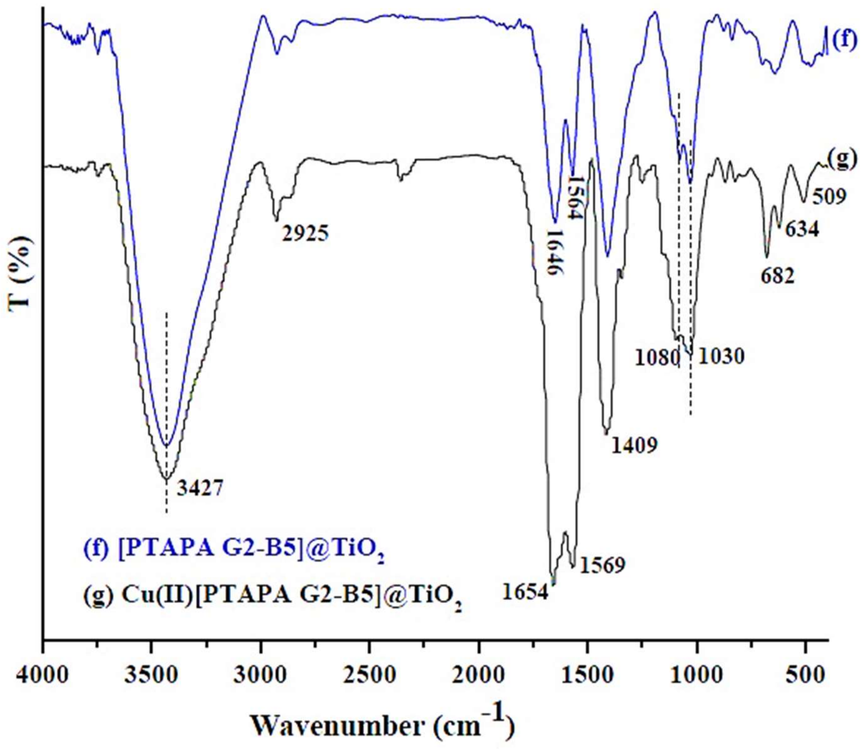

Figure S1. FT-IR spectra of (a) AP-TiO_2 (b) CC1-TiO_2 (c) Vitamin B5 (Pantothenic acid) (d) [PTAPA G2-B5]@TiO_2 (e) CC2-TiO_2 (f) [PTAPA G2-B5]@TiO_2 (g) Cu(II)[PTAPA G2-B5]@TiO_2

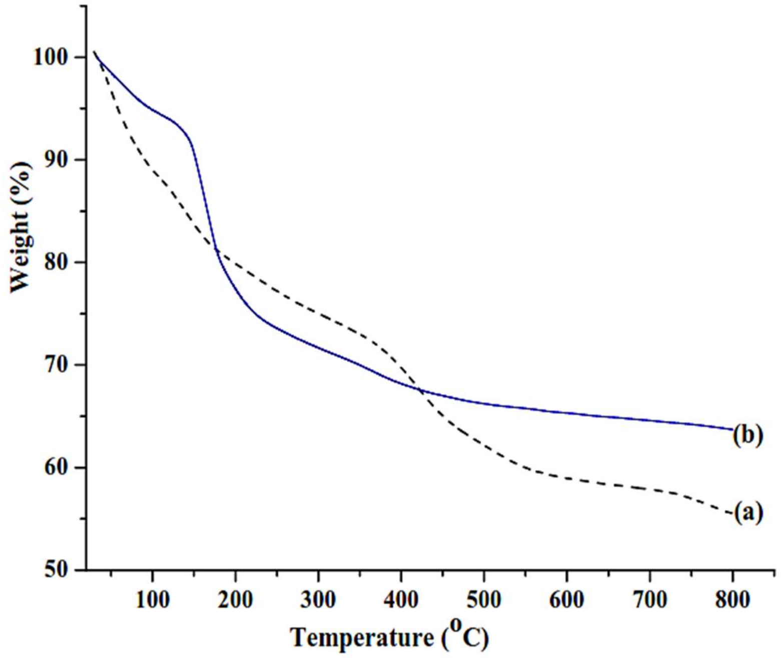

Figure S2. The TGA of (a) [PTAPA G1-B5]@TiO_2 (b) Cu(II)[PTAPA G2-B5]@TiO_2

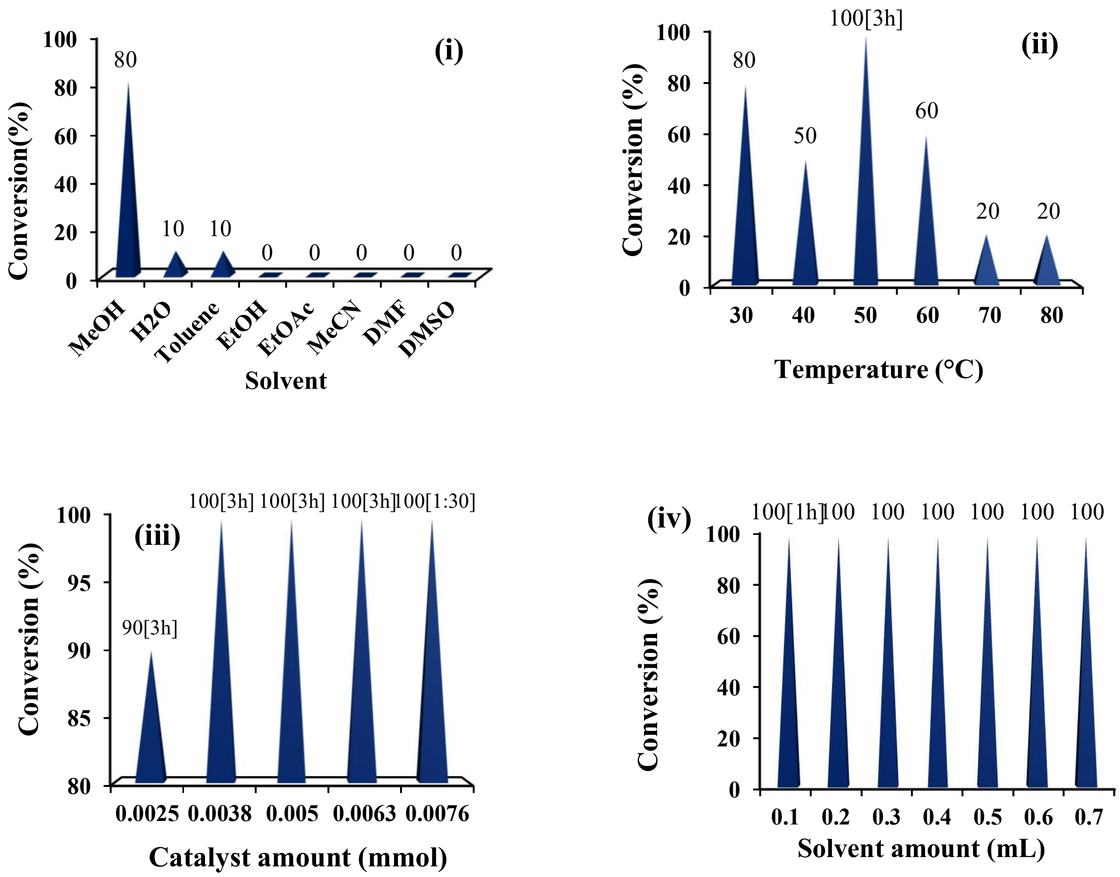

Figure S3. Optimization of the (i) solvent types [5 mol% (0.005 g, 0.006 mmol) Catalyst, RT and 0.4 mL solvent for 3h] (ii) temperature [5 mol% (0.005 g, 0.006 mmol) Catalyst and 0.4 mL MeOH for 3h] (iii) catalyst amount [50 °C and 0.5 mL MeOH] (iv) solvent amount [6 mol% (0.006 g, 0.007 mmol) Catalyst, 50 °C, and MeOH] on the homo-coupling phenylboronic acid (0.125 mmol) catalyzed by Cu (II) [PTAPA G2-B5] @TiO_2 under air, Room light (400-650 nm, 40 W).

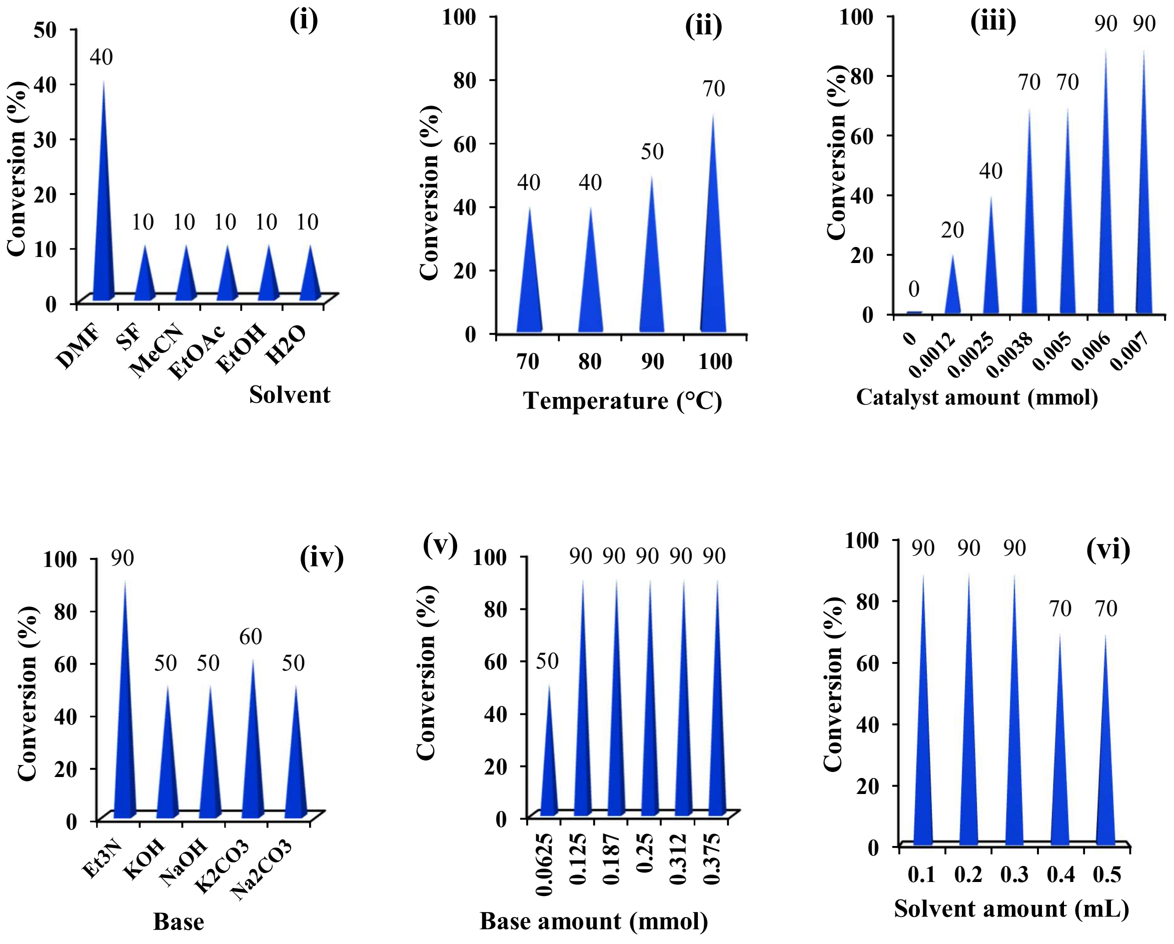

Figure S4. Optimization of the (i) solvent types [2 mol% (0.002 g, 0.0025 mmol) Catalyst, 0.125 mmol Et_3N, 80 °C and 0.2 mL solvent] (ii) temperature [2 mol% (0.002 g, 0.0025 mmol) Catalyst, 0.125 mmol Et_3N, and 0.2 mL DMF] (iii) catalyst amount [0.125 mmol Et_3N, 100 °C and 0.2 mL DMF] (iv) Base [0.125 mmol Base, 5 mol% (0.005 g, 0.006 mmol) Catalyst, 100 °C and 0.2 mL DMF] (v) Base amount [Et_3N, 5 mol% (0.005 g, 0.006 mmol) Catalyst, 100 °C and 0.2 mL DMF] and (ii) solvent amount [0.125 mmol Et_3N, 5 mol% (0.005 g, 0.006 mmol) Catalyst, 100 °C and 0.2 mL DMF] on the homo-coupling phenylacetylene (0.125 mmol) catalyzed by Cu (II) [PTAPA G2-B5]@TiO_2, Room light (400-650 nm, 40 W) after 3h.

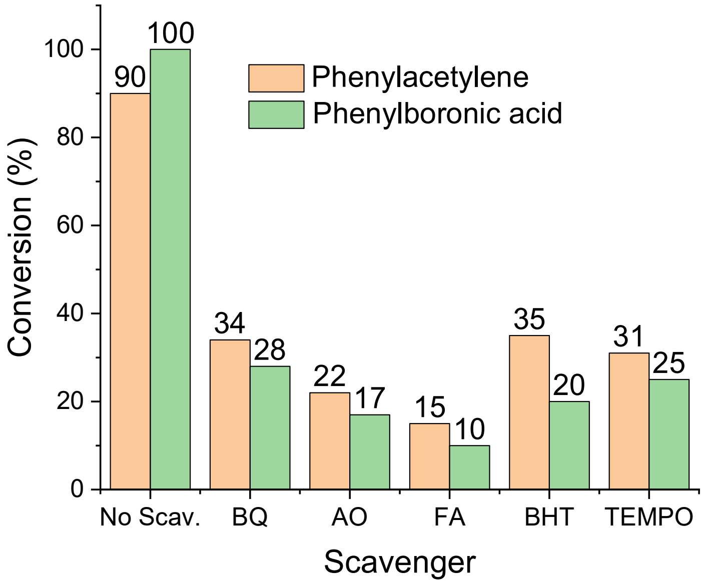

Figure S5. The effect of different scavengers (1 mol%) on the homocoupling reaction of phenylacetylene and phenylboronic acid in the presence of benzoquinone (BQ), ammonium oxalate (AO), formic acid (FA), 2,6-Di-tert-butyl-4-methylphenol (BHT) and TEMPO. For reaction conditions see the footnote of Table 1, entries 1 and 8.

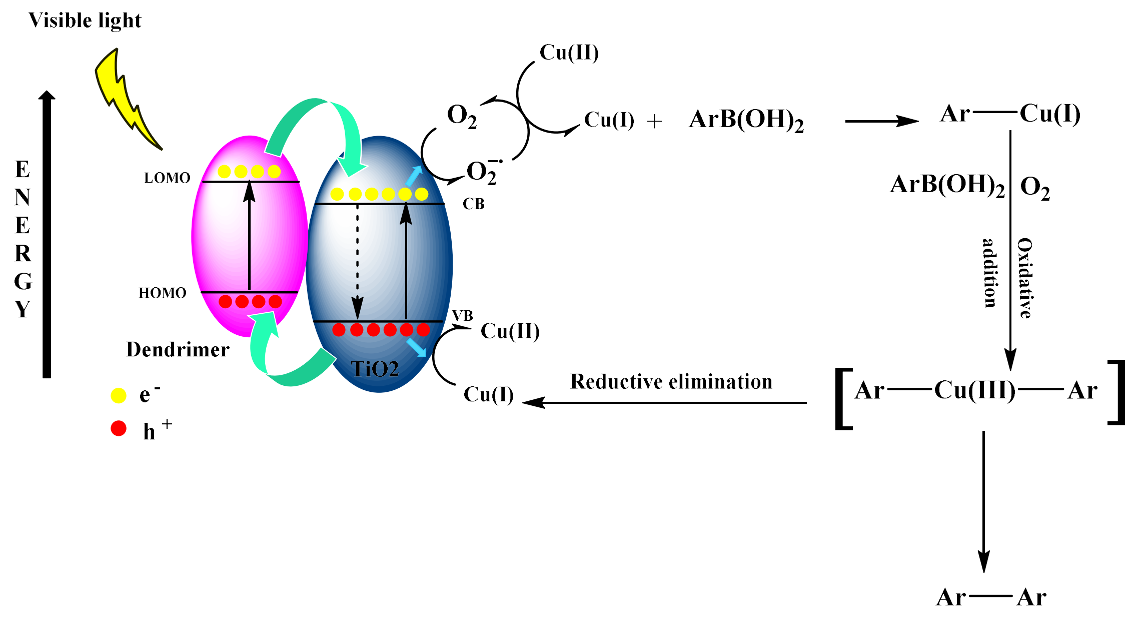

Figure S6. Proposed mechanism for homo coupling reaction of phenylboronic acid

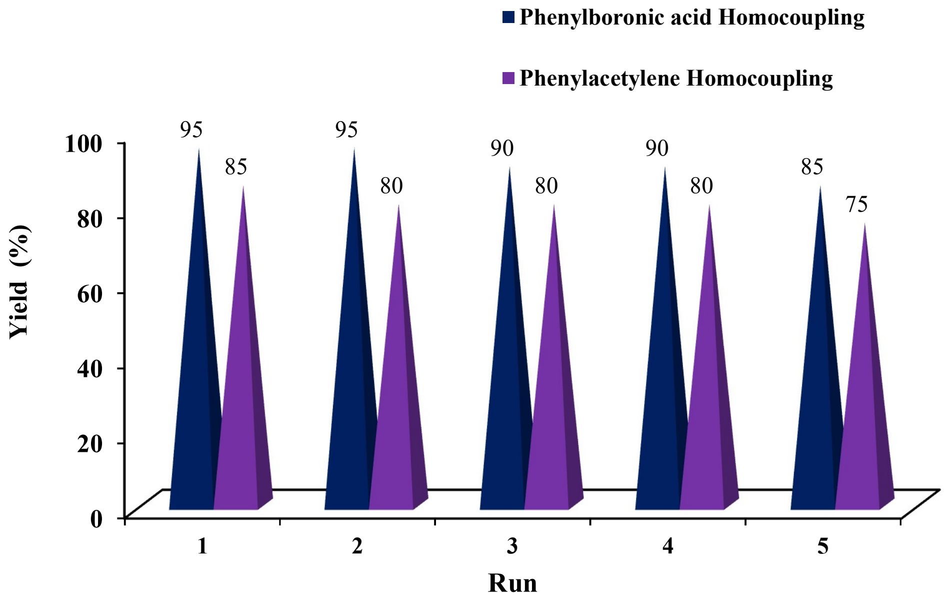

Figure S7. The catalytic activity of Cu (II) [PTAPA G2-B5] @ TiO_2 in consecutive reaction cycles of homo-coupling (a) phenylboronic acid and (b) phenylacetylene under optimized conditions.

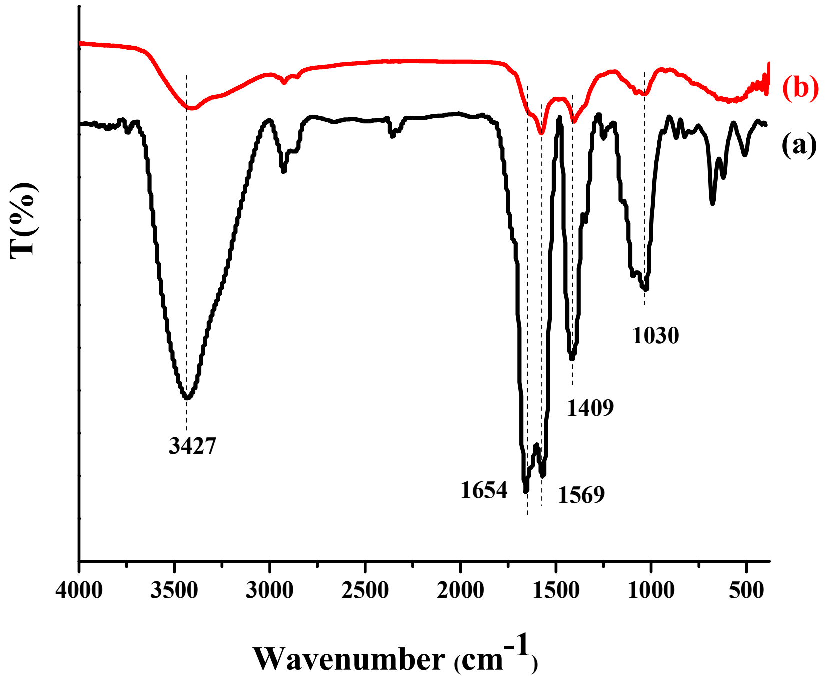

Figure S8. The FT-IR spectra of Cu (II) [PTAPA G2-B5] @ TiO_2 (a) fresh (b) after 5 times reuses of homo-coupling phenylboronic acid under optimized conditions.

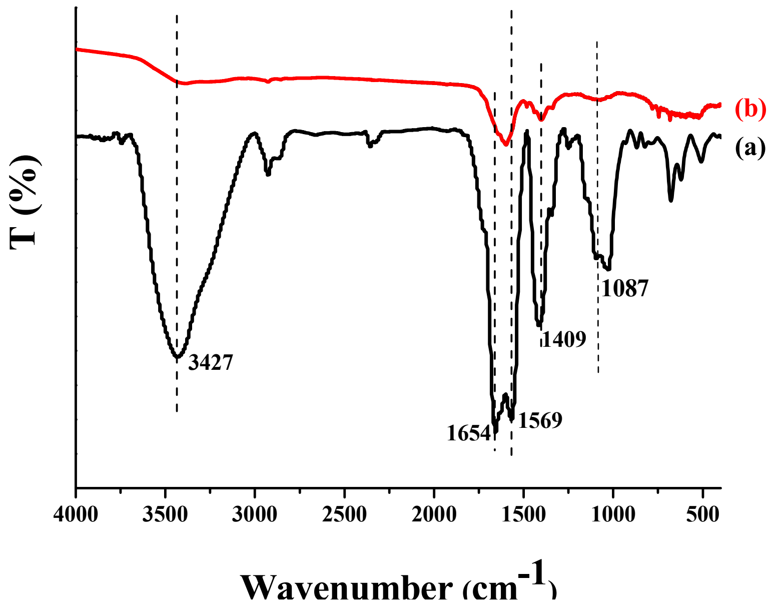

Figure S9. The FT-IR spectra of Cu (II) [PTAPA G2-B5] @ TiO_2 (a) fresh (b) after 5 times reuses of homo-coupling phenylacetylene under optimized conditions.

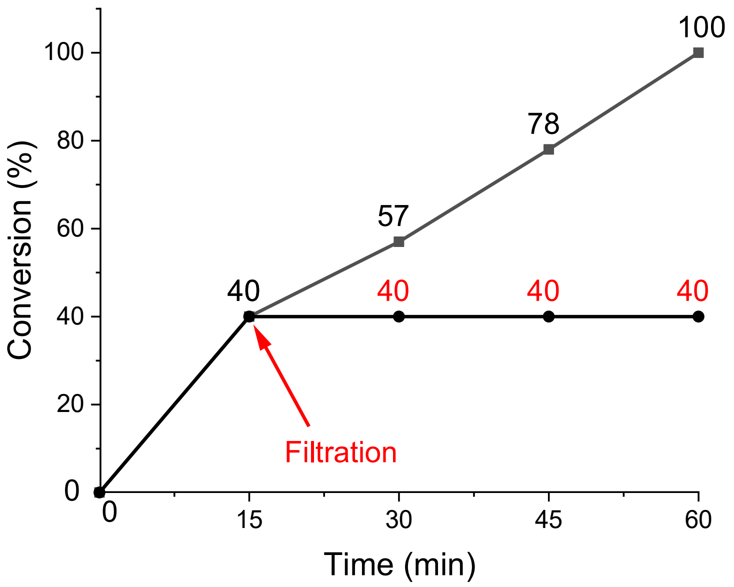

Figure S10. Hot filtration test for the homocoupling reaction of phenylboronic acid under optimized conditions (see Table 1, entry 1). Black number (in the presence of catalyst), Red numbers (after filtration).

Selected Spectra data:

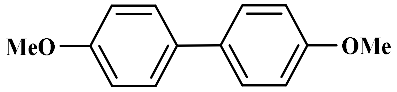

4,4'-Dimethoxybiphenyl (2): ^{1}H NMR (500 MHz, $CDCl_3$): 7.49 (d, J = 8.1 Hz, 4H), 7.01 (d, J = 8.0 Hz, 4H), 3.83 (s, 6H); ^{13}C NMR (126 MHz, $CDCl_3$): 158.6, 133.4, 127.7, 114.1, 55.3; GCMS (EI, m/z): 214 (100) [1].

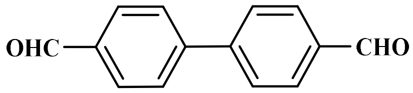

Biphenyl-4,4'-dicarbaldehyde (6): ^{1}H NMR (500 MHz, $CDCl_3$): 10.07 (s, 2H), 7.98 (d, J = 8.4 Hz, 4H), 7.78 (d, J = 8.2 Hz, 4H). ^{13}C NMR (126 MHz, $CDCl_3$): 191.6, 145.6, 135.7, 130.2, 128.1. GCMS (EI, m/z): 306 [1].

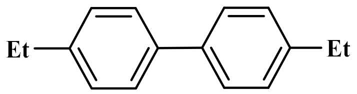

4,4'-diethyl-1,1'-biphenyl (3) ^{1}H NMR ($CDCl_3$, 400 MHz): δ 7.42– 7.40 (m, 2H, ArH), 7.30- 7.28 (m, 3H, ArH), 7.17-7.05 (m, 3H, ArH), 2.26-2.56 (q, 2H, $-CH_2$), 2.502.44 (q, 2H, $-CH_2$), 1.20 (s, 3H, $-CH_3$), 1.18 (s, 3H, $-CH_3$) [2].

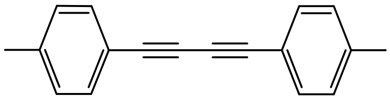

1,4-Di-p-tolylbuta-1,3-diyne: ^{1}H NMR (400.1 MHz, $CDCl_3$): δ = 7.41-7.40 (d, J = 7.9 Hz, 4H), 7.15-7.13 (d, J = 7.9 Hz, 4H), 2.36 (s, 6H) [3].

References

1. Jafarpour, M., Rezaeifard, A., Ghahramaninezhad, M. & Tabibi, T. Reusable \$α\$-MoO 3 nanobelts catalyzes the green and heterogeneous condensation of 1, 2-diamines with carbonyl compounds. *New J. Chem.* **37**, 2087–2095 (2013).
2. Karimi, B. & Safari, A. A. One-pot synthesis of α-aminonitriles using a highly efficient and recyclable silica-based scandium (III) interphase catalyst. *J. Organomet. Chem.* **693**, 2967–2970 (2008).
